# Targeting the NAT10/NPM1 axis abrogates PD-L1 expression and improves the response to immune checkpoint blockade therapy

**DOI:** 10.1186/s10020-024-00780-4

**Published:** 2024-01-20

**Authors:** Ge Qin, Fan Bai, Huabin Hu, Jianwei Zhang, Weixiang Zhan, Zehua Wu, Jianxia Li, Yang Fu, Yanhong Deng

**Affiliations:** 1 Department of General Surgery, The Sixth Affiliated Hospital, Sun Yat-Sen University, Yuan Cun Er Rd No. 26, Guangzhou, 510655 People’s Republic of China; 2 Department of Medical Oncology, The Sixth Affiliated Hospital, Sun Yat-Sen University, Yuan Cun Er Rd No. 26, Guangzhou, 510655 People’s Republic of China; 3 Guangdong Provincial Key Laboratory of Colorectal and Pelvic Floor Diseases, The Sixth Affiliated Hospital, Sun Yat-Sen University, Yuan Cun Er Rd No. 26, Guangzhou, 510655 People’s Republic of China; 4 Biomedical Innovation Center, The Sixth Affiliated Hospital, Sun Yat-Sen University, Yuan Cun Er Rd No. 26, Guangzhou, 510655 People’s Republic of China

**Keywords:** NPM1, NAT10, PD-L1, Immunotherapy, Remodelin

## Abstract

**Background:**

PD-1/PD-L1 play a crucial role as immune checkpoint inhibitors in various types of cancer. Although our previous study revealed that NPM1 was a novel transcriptional regulator of PD-L1 and stimulated the transcription of PD-L1, the underlying regulatory mechanism remains incompletely characterized.

**Methods:**

Various human cancer cell lines were used to validate the role of NPM1 in regulating the transcription of PD-L1. The acetyltransferase NAT10 was identified as a facilitator of NPM1 acetylation by coimmunoprecipitation and mass spectrometry. The potential application of combined NAT10 inhibitor and anti-CTLA4 treatment was evaluated by an animal model.

**Results:**

We demonstrated that NPM1 enhanced the transcription of PD-L1 in various types of cancer, and the acetylation of NPM1 played a vital role in this process. In particular, NAT10 facilitated the acetylation of NPM1, leading to enhanced transcription and increased expression of PD-L1. Moreover, our findings demonstrated that Remodelin, a compound that inhibits NAT10, effectively reduced NPM1 acetylation, leading to a subsequent decrease in PD-L1 expression. In vivo experiments indicated that Remodelin combined with anti-CTLA-4 therapy had a superior therapeutic effect compared with either treatment alone. Ultimately, we verified that the expression of NAT10 exhibited a positive correlation with the expression of PD-L1 in various types of tumors, serving as an indicator of unfavorable prognosis.

**Conclusion:**

This study suggests that the NAT10/NPM1 axis is a promising therapeutic target in malignant tumors.

**Supplementary Information:**

The online version contains supplementary material available at 10.1186/s10020-024-00780-4.

## Background

Immunotherapy has emerged as a promising strategy to improve the treatment outcomes of patients affected by solid tumors. Among the various immunotherapeutic approaches, blockade of immune checkpoint proteins, specifically PD-1/PD-L1 (CD279/CD274), has shown impressive effectiveness in various cancer types, including melanoma (Hamid et al. 2013), lung cancer (Reck et al. 2016), and colorectal cancer (Hu et al. 2022).

Tumors utilize the PD-1/PD-L1 pathway to suppress T-cell function, which is crucial for evading the immune response (Tumeh et al. 2014). PD-L1 is predominantly found in tumor cells, and its expression is regulated by a variety of factors (Yamaguchi et al. 2022). In general, PD-L1 can be expressed either constitutively or inducibly. The constitutive expression of PD-L1 is primarily instigated by endogenous carcinogenic alterations. For example, MYC overexpression (Casey et al. 2016) and RAS mutations exert a stimulatory effect on the expression of PD-L1 (Coelho et al. 2017). However, tumors that constitutively express PD-L1 are not sensitive to immunotherapy (Vesely et al. 2022). For inducible expression of PD-L1, interferon gamma (IFN-γ) produced by immune cells is the most effective inducer of PD-L1 expression, acting primarily through the JAK/STAT1/IRF1 signaling (Garcia-Diaz et al. 2019). Besides, epidermal growth factor (EGF) (Chen et al. 2015), interleukins (ILs) (Carbotti et al. 2021; Lu et al. 2020) and tumor necrosis factor alpha (TNF-α) (Bertrand et al. 2017) are important cytokines that stimulating PD-L1 expression. In addition, NF-κB signaling and PI3K/AKT pathways also play crucial roles in inducing PD-L1 expression (Antonangeli et al. 2020). Immune activation-induced PD-L1 expression is a crucial self-protection mechanism of tumor cells and a critical nexus for sensitivity to cancer immunotherapy. However, different tumors, and even different molecular subtypes of the same tumor type, express varied levels of PD-L1. The regulatory mechanisms of PD-L1 expression in these tumors or subtypes may be similar or distinct and have not been fully elucidated to date.

In our prior investigation, it was shown that PD-L1 expression is more elevated in triple-negative breast cancer (TNBC) than in non-TNBC. Nucleophosmin (nucleolar phosphoprotein B23, numatrin) (NPM1), a novel transcriptional regulator, attaches to the PD-L1 promoter region in TNBC cells, stimulating its transcription and inhibiting T-cell function. Moreover, this regulatory mechanism exists in murine melanoma cells, indicating that NPM1 may have analogous functions in other cancers (Qin et al. 2020).

NPM1 is extensively present and mainly found in the nucleus. It plays a vital role in chromatin remodeling, ribosome biogenesis, DNA repair, centrosome duplication, apoptosis and embryogenesis (Box et al. 2016). NPM1 can be subjected to extensive posttranslational modifications, such as phosphorylation (Shandilya et al. 2014), acetylation (Shandilya et al. 2009), ubiquitination (Nishikawa et al. 2009), and SUMOylation (Haindl et al. 2008). These modifications regulate its stability, localization, and protein interactions, as well as the associated cellular functions. In our published study, we found that some patients with positive PD-L1 expression did not exhibit high levels of NPM1 expression. Furthermore, NSC348884 (CAS: 81,624–55-7), a compound that inhibits the oligomerization of NPM1, did not exhibit the ability to effectively suppress the expression of PD-L1 (Qin et al. 2020). These findings imply that NPM1, especially monomeric NPM1, may undergo specific modifications to regulate PD-L1 expression. However, this precise modified form remains unknown.

N-acetyltransferase 10 (NAT10) has been identified as a cancer-promoting factor. It has been reported to facilitate the progression (Wang et al. 2022), metastasis (Liao et al. 2023) and chemotherapy resistance (Liu et al. 2020) in different cancers. But the role of NAT10 in tumor immunity remains unexplored and undocumented.

This study validated that NPM1 promoted the transcription of PD-L1 in different cancer types. Additionally, we demonstrated that NAT10 upregulated PD-L1 expression by acetylating NPM1. In support of this regulatory mechanism, our in vivo results showed that NAT10 deficiency increased the infiltration and activity of CD8^+^ T cells. In addition, we observed that combination therapy with a NAT10 inhibitor and an anti-CTLA-4 antibody exerted better effects than either monotherapy. Overall, our research uncovered the regulatory role of the NAT10/NPM1 axis in the transcription of PD-L1, which appears to be prevalent in different types of human malignancies. These findings emphasize the possibility of targeting NAT10/NPM1 signaling for cancer treatment.

## Methods

### Cell lines

Cell lines were sourced from iCell Bioscience Inc. (Shanghai, China). The human breast cancer cell line MDA-MB-231, human melanoma cell lines A375 and MeWo, human colorectal cancer cell lines HCT116 and SW480, mouse colorectal cancer cell line MC38 and HEK293T cell line were cultured in DMEM (Invitrogen, Carlsbad, CA, USA) containing 10% fetal bovine serum (Invitrogen), 100 U/mL penicillin and 100 μg/mL streptomycin (Invitrogen). The human breast cancer cell line MCF-7 was maintained in RPMI 1640 medium (Invitrogen) containing 10% fetal bovine serum (Invitrogen), 100 U/mL penicillin and 100 μg/mL streptomycin (Invitrogen). The cells were cultured under the conditions of 37 °C and 5% CO_2_.

### Plasmids

Plasmid vectors used in this study were obtained from Add gene (Watertown, MA, USA). The pSIN-EF2-puro vector was used to insert fragments of Flag-NPM1, V5- NAT10, and Flag-NPM1 7K-7R. The pGL3-basic plasmid was modified by inserting the PD-L1 promoter region, which extended 2000 base pairs before the transcription start site.

### Stable transduction using lentiviruses

The pLKO.1-puro vector-based lentiviral shRNA targeting mouse and human NAT10 was generated. The sh-RNA sequences were obtained from Sigma, and the clone IDs for shRNA are as follows: Human sh-NAT10-1: TRCN0000308219, sh-NAT10-2: TRCN0000296355; mouse sh-NAT10-1: TRCN0000444703, sh-NAT10-2: TRCN0000432460, sh-NAT10-3: TRCN0000446016. Stable NPM1 knockdown cells were generated by the plenti-CRISPR/Cas9-v2 system, and the sequences of the guide RNAs were as follows: sg-NPM1-1: TCACAGGTCAGTTTAGGGGC; sg-NPM1-2: ATTAGTGGACAGCACTTAGT. These lentiviral shuttle plasmids were co-transfected into the HEK293T cells with the packaging plasmids. Supernatants containing the lentivirus were collected after 48 or 72 h and then infected into cells. The infected cells were treated with puromycin for at least one week to obtain the stable cell lines.

### Western blot analysis

Cells were harvested and lysed by Cell lysis buffer (P0013, Beyotime, Shanghai, China). Protein concentration was determined by Detergent Compatible Bradford Assay Kit (23246, Invitrogen). 50–100 μg proteins were used for polyacrylamide gel electrophoresis. Subsequently, the proteins were transferred onto a PVDF membrane. We utilized primary antibodies that targeted the following proteins: NAT10 (13365–1-AP; Proteintech, Rosemont, IL, USA; 1:1,000), PD-L1 (GTX104763; GeneTex, Irvine, CA, USA; 1:2,000), PD-L1 (GTX31308; GeneTex; 1:1,000), NPM1 (FC-61991; Invitrogen, Carlsbad, CA, USA; 1:1,000), Flag (14793; Cell Signaling Technology; 1:2,000), V5 (58009; Cell Signaling Technology; 1:2,000), acetylated lysine (9441; Cell Signaling Technology; 1:1,000), HSP70 (46477; Cell Signaling Technology; 1:1,000), and GAPDH (10494–1-AP; Proteintech; 1:10,000). Every primary antibody recognized the band at the correct molecular weight. Next, membranes were covered by anti-rabbit secondary antibody (W4011, Promega, Madison, WI, USA; 1:10,000) or anti-mouse secondary antibody (RGAM001, Proteintech; 1:10,000). Finally, chemiluminescence was recorded by MiniChemi Chemiluminescent Imaging System (Sinsage Technology, Beijing, China).

### Dual luciferase reporter assay

Twenty-four-well plates were used to insert the cells. After being attached overnight, firefly luciferase reporter plasmids (0.5 μg/well) and renilla luciferase plasmids (10 ng/well) were transfected into cells. After 48 h, cells were lysed at room temperature. Luciferase signal was measured by the Dual-Luciferase Reporter Assay System Kit (E1910; Promega, Madison, WI, USA).

### RT‒qPCR assay

We used the following primers in the RT‒qPCR assay: PD-L1 forward, 5′-TGGCATTTGCTGAACGCATTT-3′; PD-L1 reverse, 5′-TGCAGCCAGGTCTAATTGTTTT-3′; NPM1 forward, 5′-GGAGGTGGTAGCAAGGTTCC-3′; NPM1 reverse, 5′-TTCACTGGCGCTTTTTCTTCA-3′; GAPDH forward, 5′-ATCACCATCTTCCAGGAGCGA-3′; and GAPDH reverse, 5′-CCTTCTCCATGGTGGTGAAGAC-3′.

### Mass spectrometry analysis

Mass spectrometry was performed as described previously (Qin et al. 2020).

### ChIP‒qPCR assay

Flag-NPM1, Flag-7K-7R NPM1, and empty vector plasmids were transfected into MDA-MB-231 cells. The ChIP experiment was conducted with the SimpleChIP@Enzymatic Chromatin IP Kit (Magnetic Beads) (9003, Cell Signaling Technology) according to the manufacturer’s protocol. Chromatin immunoprecipitation was conducted with either a control IgG or an anti-Flag primary antibody (14793; Cell Signaling Technology; 5 μg). The primers used in ChIP-qPCR are listed below: PD-L1 forward 5′-CTTCGAAACTCTTCCCGGTG-3′, reverse 5′-ACCTCTGCCCAAGGCAGCAA-3′.

### Coimmunoprecipitation (Co-IP)

Flag-NPM1 and/or V5-NAT10 plasmids were transfected into HEK293T cells. Proteins were immunoprecipitated using anti-Flag magnetic beads (B26102; Bimake, Houston, TX, USA; 10 μl). To perform coimmunoprecipitation in MDA-MB-231 cells, the lysates of the cells were treated with either a 2 μg anti-NPM1 antibody (FC-61991; Invitrogen) or a 4 μg anti-NAT10 antibody (13365–1-AP; Proteintech). Afterwards, the lysates were cultured with Protein A/G-agarose beads (B23202; Bimake, 20 μl).

### Confocal immunofluorescence microscopy

Cell samples were fixed, permeabilized and blocked. NPM1 antibody (FC-6199; Invitrogen; 1:50) and NAT10 antibody (13365–1-AP; Proteintech, 1:50) were added to the cells and incubated overnight at 4 °C. After that, the cells were exposed to secondary antibodies conjugated with Alexa Fluor™ 488 (A-11001; Invitrogen, 1:2000) and conjugated with Alexa Fluor™ 594 (A-21207; Invitrogen, 1:2000) for 30 min at ambient temperature. Cell nuclei were stained using Hoechst 33342 (62249; Invitrogen, 1:5000). Confocal immunofluorescence microscopy was conducted with LAS AF Lite.

### Animals and treatment

C57BL/6N mice, ranging in age from 4 to 6 weeks, were acquired from Beijing Vital River Laboratory Animal Technology. MC38 cells (1 × 10^6^) were subcutaneously injected. When tumors exceeded 1.5 cm in diameter, mice were sacrificed. For combination treatment, mice in the anti-CTLA-4 monotherapy group received a 200 μg/mouse dose of an anti-CTLA-4 antibody (BE0164; BioXCell, West Lebanon, NH, USA) twice weekly for a duration of two weeks. Mice in the Remodelin monotherapy group received Remodelin (S7641; Selleck Chemicals, Houston, TX, USA) at a concentration of 100 mg/kg. For a duration of 2 weeks, Remodelin was given daily through oral gavage. The mice in the group receiving combination therapy were administered the anti-CTLA-4 antibody at a dosage of 200 μg per mouse, along with Remodelin at a dosage of 100 mg/kg. The control group mice received treatment with PBS. The calculation of tumor volume (TV) was determined by using the formula TV (mm^3^) = π/6 × length × width^2^.

### Tumor-infiltrating lymphocyte (TILs) analysis

To analyze TILs, the tumor tissues were sliced into small fragments and subsequently subjected to digestion for a duration of 2.5 h using 0.6 ku/ml DNAse (D5025; Sigma) and 1 mg/ml collagenase type IV (C5138; Sigma). The cells were stained using the following antibodies: anti-CD45-FITC (11–0451-82; eBioscience; 1:100), anti-CD107a-PE (121612; Biolegend; 1:100), anti-CD8-APC (100721; Biolegend; 1:50) and anti-CD69-PE (104508; Biolegend; 1:100). All flow data were analyzed using FlowJo X.

### Human tissues and clinicopathological information

Tissue microarrays were obtained from Outdo Biotech Co., Ltd., Shanghai, China (HColA180Su21, HBreD050Bc01). These tissues were obtained from patients during their first surgery. These patients did not undergo any treatment before surgery.

### Histopathology

To perform immunohistochemical (IHC) staining, NAT10 and PD-L1 primary antibodies were diluted at a 1:100 ratio. Immunohistochemical evaluation was performed by VisioPharm software and checked by a pathologist. PD-L1 positivity was defined as a positive cell rate of ˃1%. For each sample, the extent (0–100%) and intensity (0–3 +) of NAT10 expression were assessed, and a tumor-specific overall H-score (calculated as the mean extent multiplied by intensity) was determined. An ROC curve was used to determine high and low expression of NAT10.

### Database

The sequence of the PD-L1 promoter was obtained from the UCSC database (https://genome.ucsc.edu). Correlation analysis of NPM1 and NAT10 expression with PD-L1 expression was performed in the TIMER 2.0 database (http://timer.cistrome.org/). Kaplan–Meier analysis of NAT10 expression in TNBC was performed using the Kaplan–Meier Plotter (http://www.kmplot.com).

### Data analysis

The data were analyzed and graphed using GraphPad Prism 6.0 and SPSS. Student's t tests were used for comparisons between two groups, and one-way ANOVAs were used for comparisons between multiple groups. Kaplan–Meier method and Cox regression analysis were used for survival analysis. Pearson's chi-square test was used for correlation analysis. P < 0.05 was considered statistically significant.

## Results

### NPM1 promotes PD-L1 transcription and expression in multiple cancer cells

**Fig. 1:**
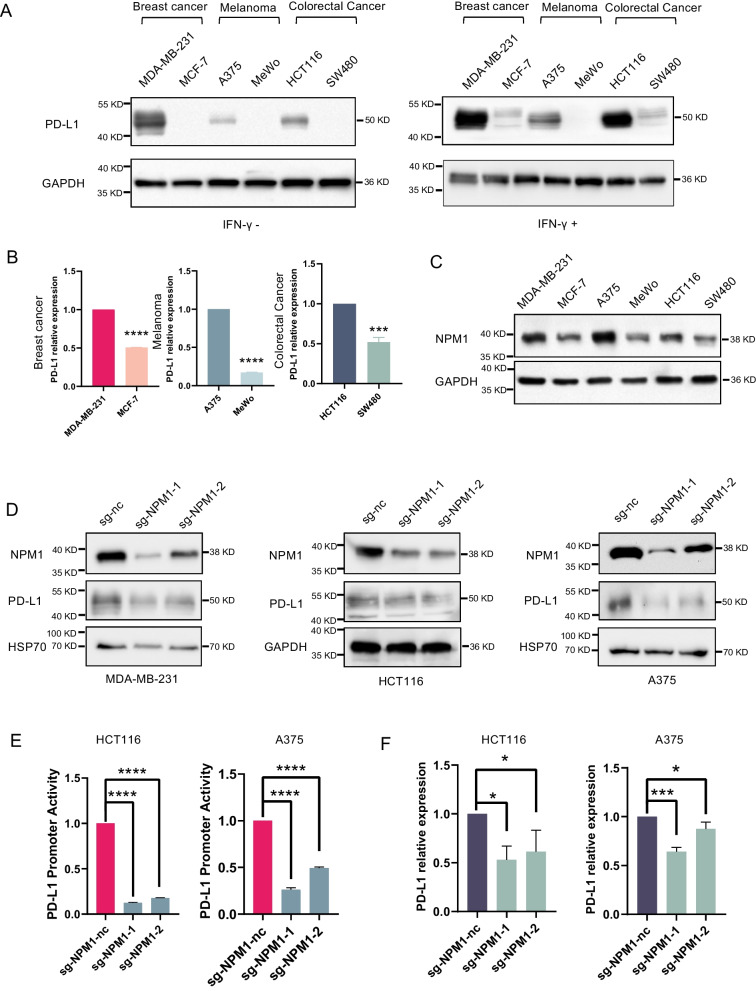
NPM1 transcriptionally promotes PD-L1 expression in different cancer cell lines. **A** The indicated cells were treated with (right) or without (left) 25 ng/ml IFN-γ for 24 h. **B** PD-L1 mRNA levels in the indicated cell lines were measured by qPCR and normalized fold change values were shown. Gene expression levels of PD-L1 high expression were set at 1.0. **C** Western blot analysis of NPM1 in the indicated cell lines. **D** NPM1 was stably knocked down by sgRNAs in MDA-MB-231, HCT116 and A375 cells. The expression of NPM1 and PD-L1 was evaluated. **E**, **F** PD-L1 mRNA levels were measured by qPCR (**F**), and promoter activity was measured by a dual-luciferase reporter assay (**E**). Data are presented as the mean ± s.d. of three independent experiments, ***P < 0.001, ****P < 0.0001

Numerous studies have indicated that the expression of PD-L1 is significantly elevated in tumor tissues compared to normal tissues (Geng et al. 2008; Dong et al. 2002). In our previous research, a critical role for NPM1 in the transcription of PD-L1 has been identified in TNBC cells, which exhibit elevated levels of PD-L1 expression (Qin et al. 2020). Moreover, through database mining, it was found that PD-L1 and NPM1 expression were positively correlated in both colon cancer and skin cutaneous melanoma (Additional file 1: Fig. S1A). Therefore, we speculated that this regulatory mechanism of PD-L1 might be present in different types of carcinomas. First, the status of PD-L1 was determined in various types of cancer cells. MDA-MB-231 is a TNBC cell line, and MCF-7 is a non-TNBC cell line. A375 is a skin melanoma cell line with the BRAF-V600E mutation, while MeWo cells harbor wild-type BRAF. HCT116 is a microsatellite-instable colorectal cancer cell line; by contrast, SW480 cells are microsatellite-stable. Among them, MDA-MB-231, A375 and HCT116, which had comparatively high protein and mRNA levels of PD-L1, exhibited further induction of PD-L1 expression after IFN-γ treatment (Fig. 1A, B). Consistently, the MDA-MB-231, A375 and HCT116 cells expressed comparatively high levels of NPM1 (Fig. 1C).

Then, cells were stably infected with sgRNAs targeting the NPM1 gene. As illustrated in Fig. 1D–F, PD-L1 expression and promoter activity were reduced in different cell lines by NPM1 depletion. Collectively, these results suggested that NPM1 was a crucial regulator of PD-L1 transcription and expression in multiple cancers.

### Acetylated NPM1 stimulates PD-L1 transcription

**Fig. 2:**
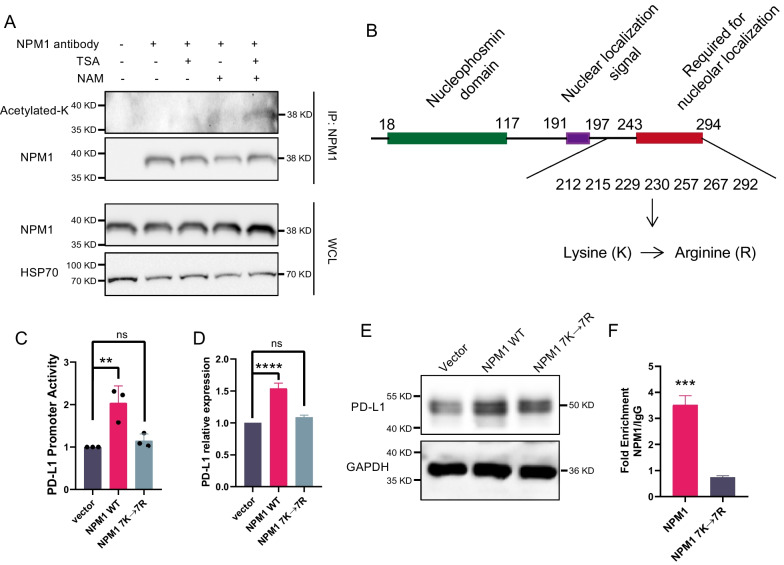
Acetylation-defective NPM1 fails to promote PD-L1 transcription and expression. **A** MDA-MB-231 cells were treated with trichostatin A (TSA, 5 μmol/L), nicotinamide (NAM, 5 mmol/L) or TSA and NAM for 12 h, and lysates were then subjected to immunoprecipitation with an anti-NPM1 antibody. **B** Schematic representation of the 7K-7R mutation in NPM1. **C**–**E** Vector, NPM1 wild-type overexpression plasmid (NPM1 WT) or NPM1 7K-7R mutant plasmid (NPM1 7 K-7R) was transfected into MDA-MB-231 cells. The promoter activity (**C**), mRNA level (**D**) and protein expression level (**E**) of PD-L1 were measured. **F** The Flag-NPM1 or Flag-NPM1 7K-7R mutant plasmid was transfected into MDA-MB-231 cells. A ChIP assay was performed with an anti-Flag antibody. Data are presented as the mean ± s.d. of three independent experiments. **P < 0.01; ***P < 0.001; ****P < 0.0001; ns, not significantly different

Since acetylation of NPM1 is important for its function (Shandilya et al. 2009), it was detected in MDA-MB-231 cells (Fig. 2A). K292, K267, K257, K230, K229, K215 and K212 are reported to be the main acetylation sites of NPM1 (Shandilya et al. 2009; Gadad et al. 2011). Therefore, Flag-tagged 7K-7R mutant and wild-type (WT) NPM1 constructs were generated (Fig. 2B). Transfection of WT NPM1 but not the 7K-7R mutant stimulated the promoter activity and expression of PD-L1 (Fig. 2C–E). Furthermore, the ChIP‒qPCR assay showed that the 7K-7R mutant disrupted the binding of NPM1 to the PD-L1 promoter (Fig. 2F). In addition, we detected the acetylation of K267 and K292 sites in mass spectrometry (Additional file 1: Fig. S2A). These two sites of NPM1 were then mutated simultaneously. Despite a decrease, this double-site mutant maintained its activity in promoting PD-L1 expression (Additional file 1: Fig. S2B). It was implied that the seven acetylation sites noted above may have a pivotal function. The above results indicated that acetylation-defective NPM1 was incapable of activating PD-L1 transcription.

### NAT10 interacts with NPM1 and promotes its acetylation

**Fig. 3:**
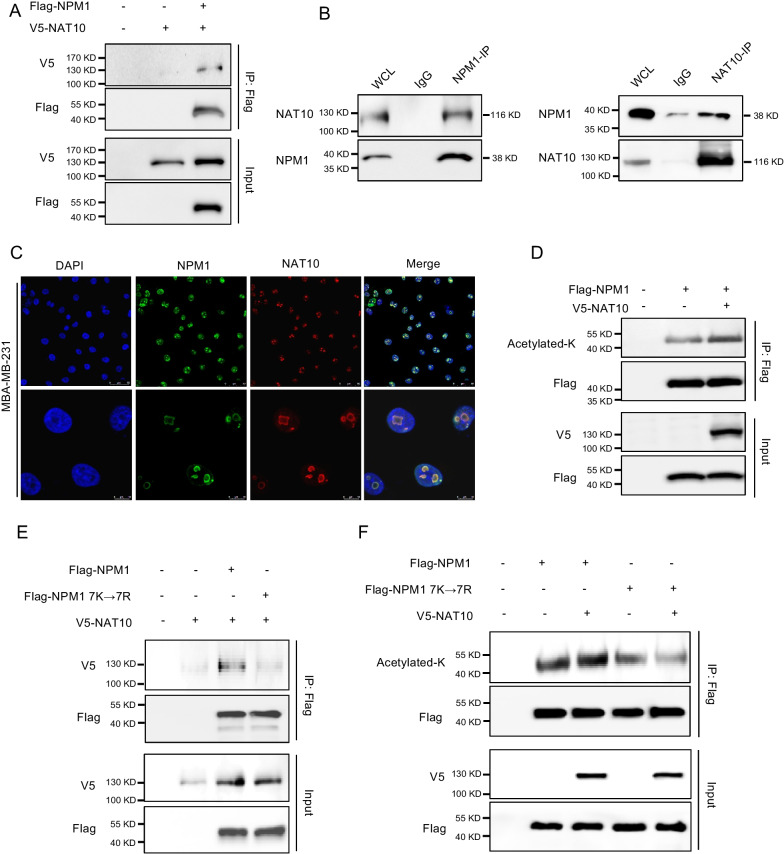
NAT10 interacted with and acetylated NPM1. **A** HEK293T cells were transfected with Flag-NPM1 and/or V5-NAT10. Cell lysates were immunoprecipitated with an anti-Flag antibody. **B** MDA-MB-231 cell lysates were immunoprecipitated with an anti-NPM1 antibody (left), an anti-NAT10 antibody (right), or control IgG. **C** Localization of NPM1 and NAT10 in MDA-MB-231 cells was evaluated by IF. **D** HEK293T cells were transfected with Flag-NPM1 and/or V5-NAT10. Cell lysates were immunoprecipitated with an anti-Flag antibody. **E**, **F** Flag-NPM1 or Flag-7K-7R was cotransfected with V5-NAT10 into HEK293T cells. Cell lysates were immunoprecipitated with anti-Flag antibody

Then, we utilized coimmunoprecipitation and mass spectrometry to identify proteins that interact with NPM1. Intriguingly, NAT10, a well-known acetyltransferase, was among the candidate proteins (Additional file 1: Fig. S1B). The interaction of NAT10 and NPM1 was proven by immunoprecipitation (Fig. 3A, B). Immunofluorescence (IF) staining showed that NAT10 and NPM1 were colocalized in the nuclei (Fig. 3C and Additional file 1: Fig. S1C). Additionally, NAT10 overexpression effectively increased the acetylation level of NPM1 (Fig. 3D). However, the NPM1 7K-7R mutant was incapable of interacting with NAT10 (Fig. 3E). Furthermore, NAT10 overexpression enhanced the acetylation level of WT NPM1 but not the NPM1 7 K-7R mutant (Fig. 3F). These results demonstrated that NPM1 was acetylated by NAT10.

### The transcription of PD-L1 is facilitated by NAT10

**Fig. 4:**
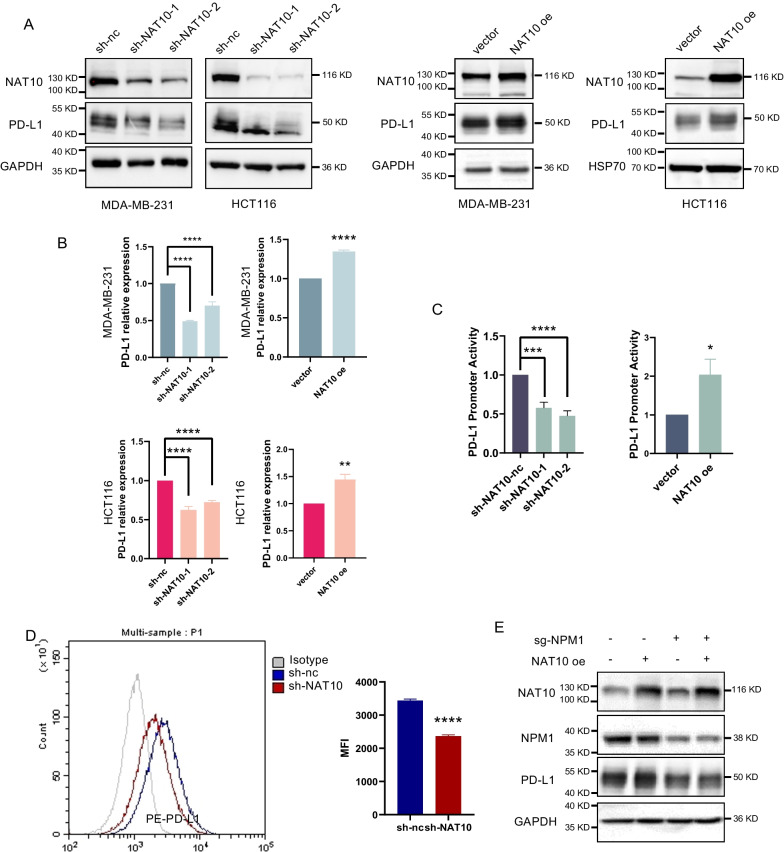
NAT10 promotes PD-L1 transcription in an NPM1-dependent manner. **A** PD-L1 protein expression in the indicated cells transfected with control shRNA (nc), NAT10 shRNAs (sh-NAT10-1, sh-NAT10-2), empty vector, or the NAT10 overexpression plasmid (NAT10 oe). **B** PD-L1 mRNA levels were quantified by RT‒qPCR. **C** PD-L1 promoter activity was quantified by a dual-luciferase assay. **D** Cell surface PD-L1 expression on MDA-MB-231 cells was examined by flow cytometry (left), and the mean fluorescence intensity (MFI) of PD-L1 was measured (right). **E** MDA-MB-231 cells with stable knockdown of NPM1 were transiently transfected with or without the NAT10 overexpression plasmid. Data are presented as the mean ± s.d. of three independent experiments. *P < 0.05, **P < 0.01; ***P < 0.001; ****P < 0.0001; ns, not significantly different

We additionally validated the involvement of NAT10 in the regulation of PD-L1 transcription. As shown in Fig. 4A–C and Additional file 1: Fig. S1E, NAT10 deficiency decreased PD-L1 expression and promoter activity, while NAT10 overexpression had the opposite effect. NAT10 knockdown also decreased PD-L1 level with the presence of IFN-γ (Additional file 1: Fig. S1D). In addition, cells with silenced NAT10 exhibited lower levels of PD-L1 expression on their cellular membrane (Fig. 4D). Next, NAT10 was overexpressed in NPM1-deficient MDA-MB-231 cells. Notably, the augmented PD-L1 expression resulting from NAT10 overexpression was completely eliminated when NPM1 was not present (Fig. 4E). These findings revealed that NAT10 activated PD-L1 transcription and expression in an NPM1-dependent manner.

### Knockdown of NAT10 increases the infiltration and activation of CD8^+^ T-cells

**Fig. 5:**
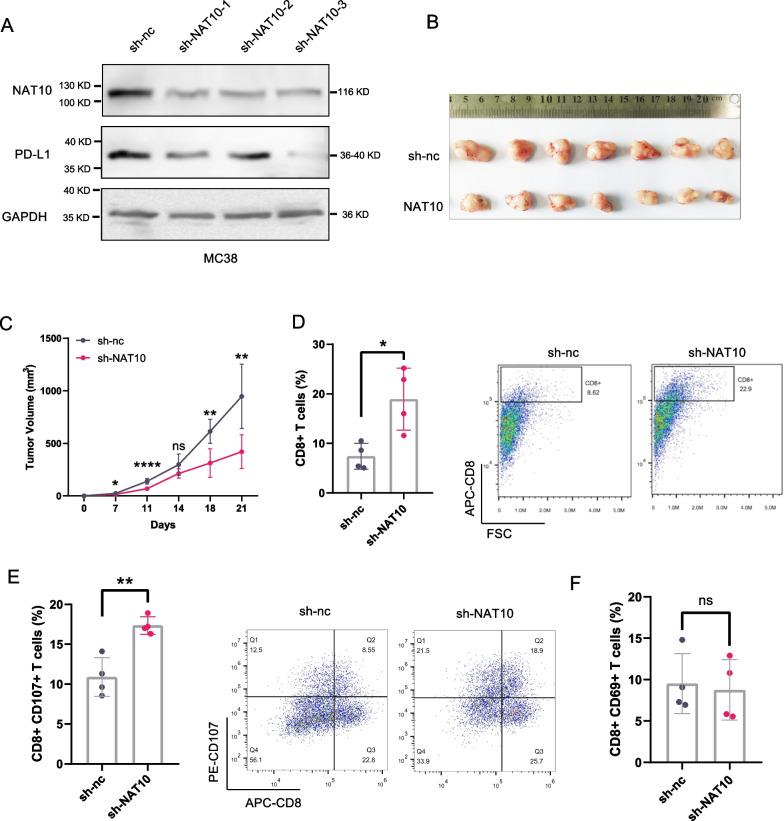
NAT10 modulated CD8 + T-cell infiltration and activity in vivo. **A** MC38 cells were stably transfected with control shRNA (sh-nc) or NAT10 shRNA. NAT10 and PD-L1 expression was measured by western blotting. **B** Pictures of the tumors excised after 21 days (n = 7). **C** The average tumor volume of each group over time is shown. **D**–**F** TILs in the tumors of each group (n = 4) were analyzed by flow cytometry. CD8^+^ cells (**D**), CD107^+^CD8^+^ cells (**E**) and CD69^+^CD8^+^ cells (F) were gated on CD45. Data are presented as the mean ± s.d. *P < 0.05, **P < 0.01; ***P < 0.001; ****P < 0.0001; ns, not significantly different

In order to confirm the role of NAT10 in modulating the tumor microenvironment in vivo, stable NAT10 knockdown MC38 cells were generated (Bao et al. 2023; Juneja et al. 2017). Consistent with the above findings, NAT10 knockdown reduced PD-L1 level in MC38 mouse colon cancer cells (Fig. 5A). MC38 cells stably transfected with sh-NAT10-3 were injected to C57/BL6N mice subcutaneously. In vivo experiments showed that knockdown of NAT10 markedly suppressed tumor growth (Fig. 5B, C). Next, we examined TILs and observed an increase in the CD8^+^ T-cell population in tumors with NAT10 knockdown (Fig. 5D, Additional file 1: Fig. S2C). Furthermore, the proportion of CD8^+^ T cells expressing CD107a, a marker for cytotoxic T-cell degranulation, was significantly increased in NAT10-silenced tumors (Fig. 5E). Nevertheless, there was no significant alteration in the percentage of CD8^+^ T cells expressing CD69, which serves as an early marker for T-cell activation (Fig. 5F). These data supported the idea that NAT10 inhibited the infiltration and function of T-cells within the tumor microenvironment.

### Treatment with a NAT10 inhibitor combined with an anti-CTLA-4 antibody has a better effect than either monotherapy

**Fig. 6:**
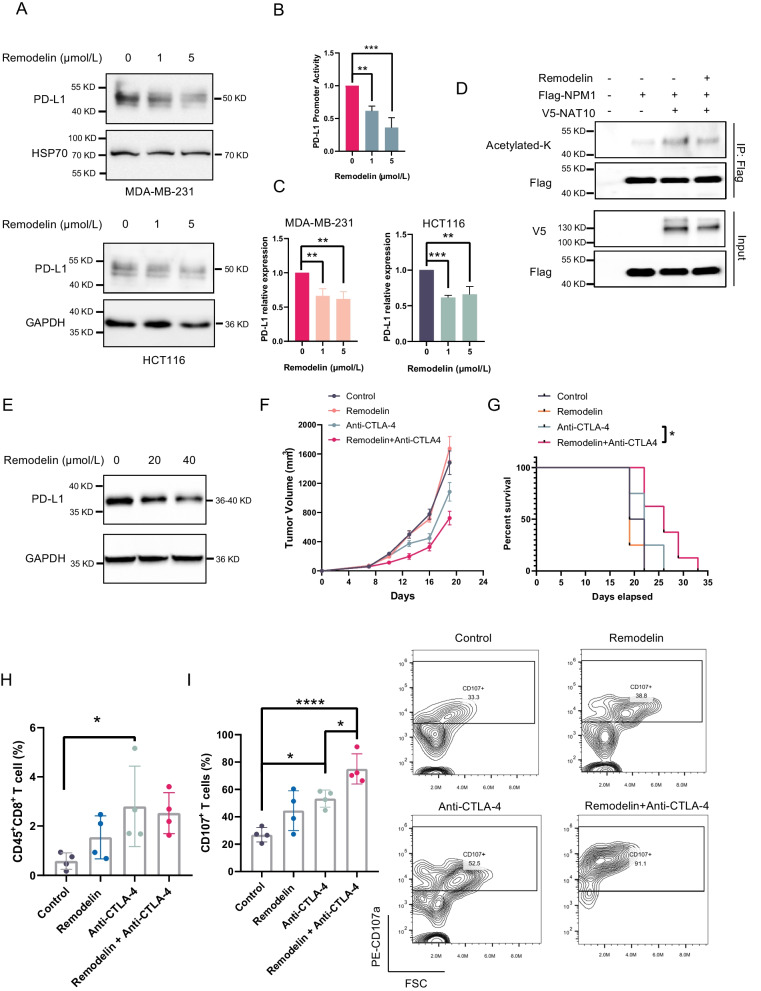
Remodelin combined with anti-CTLA-4 antibody treatment provided better effects than either monotherapy in vivo*.* **A** After cells were treated with the indicated concentration of Remodelin for 48 h, PD-L1 expression was evaluated. **B** PD-L1 promoter activity in MDA-MB-231 cells was measured after Remodelin treatment. **C** The PD-L1 mRNA level was examined after Remodelin treatment. **D** HEK293T cells were cotransfected with Flag-NPM1 and V5-NAT10 and then treated with or without 5 μmol/L Remodelin for 48 h. Cell lysates were immunoprecipitated with an anti-Flag antibody. **E** PD-L1 expression in the MC38 cell line was evaluated following Remodelin treatment. **F** The average tumor volume of each group over time is shown (n = 8). **G** Survival curves for different groups of mice (n = 8). **H**, **I** Tumor-infiltrating CD8^+^ T cells (H) and CD8^+^CD107a^+^ T cells (I) of each group (n = 4) were analyzed by flow cytometry. *P < 0.05, **P < 0.01, ***P < 0.001, ****P < 0.0001

The above data established that NAT10 promoted PD-L1 expression by acetylating NPM1, indicating that targeting NAT10 might decrease PD-L1 expression and cooperate with other immunotherapies. Remodelin, a NAT10 inhibitor, was used to target NAT10. Remodelin was found to decrease PD-L1 expression and promoter activity, as demonstrated in Fig. 6A–C. Consistent with our previous findings, Remodelin treatment also effectively inhibited NPM1 acetylation (Fig. 6D).

The combination of PD-1/PD-L1 inhibitors and anti-CTLA-4 antibody treatment has been shown to be therapeutically effective in numerous studies (Rotte 2019). Based on our findings that inhibition of NAT10 can decrease PD-L1 expression, we speculated that the combination of Remodelin with an anti-CTLA-4 antibody would also be therapeutically effective. Thus, mice bearing MC38 tumors were treated with Remodelin and/or anti-CTLA-4. The results showed that Remodelin and anti-CTLA-4 combination therapy obviously prolonged survival (Fig. 6E-G). Although not statistically significant, the mean tumor volume was smaller in the combination therapy group (Fig. 6F, Additional file 1: Fig. S2D). Of note, the percentage of CD8^+^ T cells was not apparently increased, but the proportion of CD107a^+^ T cells among CD8^+^ T cells was dramatically increased in tumors receiving the combination therapy (Fig. 6H, I). Overall, these results indicated that Remodelin combined with anti-CTLA-4 antibody treatment provided better effects than either monotherapy in vivo.

### NAT10 expression is positively associated with PD-L1 and serves as an unfavorable prognostic factor in various cancer types

**Fig. 7:**
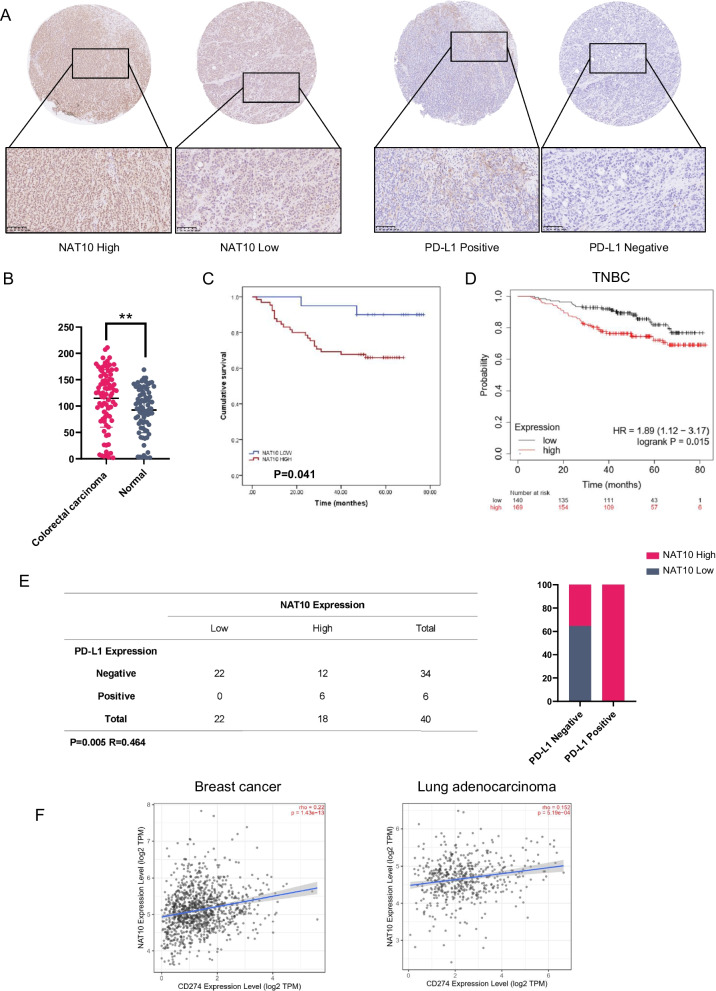
NAT10 expression positively correlates with PD-L1 expression and is associated with poor prognosis. **A** Representative images of IHC staining of NAT10 (left) and PD-L1 (right) in colon cancer tissues. **B** H-scores of NAT10 staining in human colon cancer tissues and normal adjacent tissues. Data are presented as the mean ± s.d. **P < 0.01. **C** Kaplan‒Meier analysis based on NAT10 expression in colon cancer patients. Data were analyzed by the two-sided log rank test, *P* = 0.041. **D** Kaplan‒Meier analysis based on NAT10 expression in TNBC patients using the Kaplan‒Meier Plotter database. **E** Correlation analysis between NAT10 expression and PD-L1 expression in 40 TNBC patients. Two-tailed Pearson’s chi-square test was used to determine the correlation. **F** The correlation between NAT10 and PD-L1 (CD274) expression levels in breast cancer and lung adenocarcinoma in the TIMER 2.0 database

To further validate the prognostic value of NAT10 and the correlation of its expression with PD-L1, immunohistochemical staining was conducted on tissue microarrays comprising 85 colon cancer specimens and 40 TNBC specimens (Fig. 7A and Additional file 1: Fig. S3A). In comparison to the adjacent normal tissues, the expression of NAT10 in colon tumor tissues was significantly increased (Fig. 7B). Although NAT10 expression did not appear to be an independent prognostic factor according to Cox regression analysis (Additional file 1: Table S1), the Kaplan‒Meier curve indicated that individuals with elevated NAT10 expression levels experienced reduced overall survival rates among colon cancer patients (Fig. 7C). Furthermore, high expression of NAT10 was linked to late clinical stage in colon cancer (Additional file 1: Table S2). Owing to the lack of prognostic data for the patients represented in our TNBC tissue microarray, we analyzed the prognostic value of NAT10 in TNBC by utilizing Kaplan‒Meier Plotter. Consistently, the database analysis suggested that NAT10 was a factor indicating poor prognosis in TNBC (Fig. 7D).

Moreover, in TNBC patients, NAT10 expression showed a positive correlation with PD-L1 (Fig. 7E). In addition, we observed a trend toward a higher incidence of high NAT10 expression among colon cancer patients with positive PD-L1 expression, despite the lack of statistical significance (Additional file 1: Fig. S3B). The correlation between NAT10 and PD-L1 expression was confirmed by the TIMER database. A positive correlation between PD-L1 and NAT10 expression was found in both breast cancer and lung adenocarcinoma (Fig. 7F).

## Discussion

NPM1 was identified as a novel transcriptional regulator of PD-L1 in TNBC in our previous study. However, this regulatory mechanism may not be limited to TNBC. In this study, we proved that PD-L1 transcription was activated by NPM1 in melanoma and colorectal cancer cells as well. However, the potential mechanism by which NPM1 transcriptionally regulates PD-L1 remains unclear. It is known that the activity of NPM1 is regulated by multiple posttranslational modifications. Therefore, we speculated that these posttranslational modifications may regulate the transcriptional activity of NPM1.

Phosphorylation and acetylation appear to be the most frequent modifications of NPM1. Phosphorylation of NPM1 is considered to be essential to preserve its protein binding functionality (Koike et al. 2010), uncoiling of sperm DNA (Okuwaki et al. 2012) and facilitation of mitosis (Shandilya et al. 2014). However, the phosphorylation sites in NPM1 and their functions are complicated (Sridharan et al. 2022; Wiesmann et al. 2019). Phosphorylation of Ser254 and Ser260 lowers the partitioning of NPM1 to the nucleolus and decreases the RNA and protein interactions of NPM1 (Sridharan et al. 2022). Phosphorylation of NPM1 at Ser10 and Ser70 controls cell cycle progression through the G2/M transition by modulating Cdk1 and Cdc25C activity (Du et al. 2010). According to the available studies, acetylation may play a more critical role than phosphorylation in regulating the transcriptional regulatory function of NPM1. For instance, acetylated NPM1 has been reported to transcriptionally regulate certain genes associated with oral cancer and to localize at their promoters (Shandilya et al. 2009; Senapati et al. 2022). NPM1 has also been demonstrated to act as a histone chaperone in humans. Acetylated NPM1 interacts with core histones and enhances acetylation-dependent chromatin transcription, thus leading to transcriptional activation (Swaminathan et al. 2005).

Therefore, we showed that acetylation-defective NPM1 failed to promote PD-L1 expression. Next, NAT10 was identified to interact with and acetylate NPM1. NAT10 is known as an acetyltransferase for N4-acetylcytidine (ac4C) mRNA modification. It catalyzes mRNA acetylation to enhance mRNA stability and translation efficiency (Jin et al. 2020). However, many studies have demonstrated that NAT10 is a protein acetyltransferase. For instance, p53 is acetylated and stabilized by NAT10 at K120 to inhibit cell proliferation in colorectal cancer (Liu et al. 2016). In HeLa cells, NAT10 and SIRT1 regulate centrosome duplication by mediating the acetylation of CCDC84 (Wang et al. 2019). MORC2 is acetylated by NAT10 and contributes to cell cycle activation (Liu et al. 2020). Similar to these findings, our results indicated that NAT10 functions as a lysine acetyltransferase of NPM1.

Our in vivo experiments revealed that knockdown of NAT10 drastically suppressed tumor growth and increased the infiltration and activity of CD8^+^ T cells. NAT10 is involved in multiple cancers and performs a tumor-promoting function. For instance, NAT10 facilitates gastric cancer metastasis through N4-acetylated COL5A1 (Zhang et al. 2021). In addition, NAT10 contributes to bladder cancer progression by mediating N4-acetylcytidine modification of mRNA (Wang et al. 2022). In colorectal cancer, NAT10 facilitates tumor progression by ac4C modification of KIF23 mRNA (Jin et al. 2022). To date, there has been no evidence that NAT10 promotes tumor growth by regulating immune checkpoint molecules and influencing T-cell infiltration. Our study confirmed the role of NAT10 in regulating PD-L1 transcription and immunosuppression.

To suppress NAT10 activity, we used Remodelin, a small molecule inhibitor of NAT10. Remodelin was originally discovered to overcome accelerated aging syndromes in humans (Larrieu et al. 2014). In tumors, Remodelin was reported to significantly inhibit tumor growth and tumor cell proliferation in a PDX model of head and neck squamous cell carcinoma (HNSCC) (Tao et al. 2021). Remodelin also effectively inhibits prostate cancer cell growth (Ma et al. 2022). In contrast to these studies, our in vivo experiment did not show a significant tumor-suppressive effect of Remodelin monotherapy. However, Remodelin has the potential to be combined with immunotherapy because of its regulatory activity on PD-L1. Our in vivo data showed that the combination of Remodelin and an anti-CTLA-4 antibody had a more effective therapeutic outcome than either monotherapy. There are reports of other small molecular compounds enhancing the antitumor effects of immunotherapy. For example, amlexanox, a drug used to treat asthma, has been found to synergize with anti-PD-1 mAb by inducing PD-L1 expression on dendritic cells (Takeda et al. 2021).

NAT10 and NPM1 colocalize and interact with each other predominantly within nuclei. Consequently, NAT10 expression in tumor tissues was scored by the intensity of staining in the nucleus. NAT10 expression was significantly elevated in tumor tissues and was associated with shorter survival in colon cancer patients (Fig. 7A–C). Furthermore, NAT10 expression showed an apparent positive association with PD-L1 expression in TNBC (Fig. 7E). However, we did not observe a significant association between the expression of NAT10 and PD-L1 in patients with colon cancer (Additional file 1: Fig. S3A, B). This could be due to differences in the molecular subtypes of colon cancer (e.g., RAS mutation, BRAF mutation and microsatellite instability status). These subtypes exhibit distinct biological behaviors, clinical characteristics, driver genes and therapeutic responses. The role of NAT10 in regulating PD-L1 expression may vary among them. These issues will be addressed in our future research.

There were limitations in our study. Firstly, we identified seven known acetylation sites that appear to play a pivotal role in NPM1’s ability to stimulate PD-L1 transcription. However, we were unable to confirm the minimum number of acetylations required for NPM1 function. Secondly, it is possible that other acetyltransferases, such as p300, may mediate NPM1 acetylation. The potential for these acetyltransferases to regulate PD-L1 expression by acetylating NPM1 was not clarified in our study.

## Conclusions

In conclusion, we proved that the function of NPM1 in promoting PD-L1 transcription was widely detected in multiple solid tumor types. Notably, acetylated NPM1 played a key role in activating PD-L1 transcription. NAT10 acetylated NPM1 to promote PD-L1 transcription and subsequently suppressed CD8^+^ T-cell infiltration and activity. Importantly, this regulatory mechanism establishes a basis for combination therapy with anti-CTLA-4 antibodies and NAT10 inhibitors in cancer. Therefore, our research indicated that the NAT10/NPM1 axis could be a promising target for cancer treatment.

### Supplementary Information

**Additional file 1**: **Figure S1.** **A** The correlation between NPM1 and PD-L1 (CD274) expression levels in colon cancer and skin cutaneous melanoma in the TIMER 2.0 database. **B** NAT10 was identified by mass spectrometry. **C **NPM1 and NAT10 loci were evaluated by IF staining in HCT116 cells. **D** MDA-MB-231 and HCT116 cells were treated with 25 ng/ml IFN-γ for 24 h, and PD-L1 expression was subsequently evaluated. **E** PD-L1 expression was measured by western blot (left) and qPCR (right) after NAT10 was knocked down by siRNA in A375 cell. Data are presented as the mean ± s.d. of three independent experiments. ****P < 0.0001. **Figure S2. A** The acetylation sites of NPM1 were detected by mass spectrometry. **B** PD-L1 expression was measured by western blot (right) and qPCR (left) after the indicated plasmids were transfected into MDA-MB-231 cell. **C** Gating strategies used for flow cytometric analyses in mouse tumor tissues. **D** The tumor volume of every mouse in each group (n = 8) was recorded twice a week. Data are presented as the mean ± s.d. of three independent experiments. *P < 0.05, **P < 0.01; ns, not significantly different. **Figure S3. A** Representative images of IHC staining of NAT10 (left) and PD-L1 (right) in 40 TNBC patient tissues. **B** Correlation analysis between NAT10 expression and PD-L1 expression was performed in 85 colon cancer patients using two-tailed Pearson’s chi-square test. (C) PDL1 expression was measured by western blot after HCT116 cells were treated with CPTH2 for 48 h. **Table S1.** Multivariate analysis for OS in 85 colon cancer patients. **Table S2.** Correlation analysis of NAT10 expression and clinical features in 85 colon cancer patients.

## Data Availability

The authors confirm that the data supporting the findings of this study are available within the article and its Additional materials.

## Abbreviations

NPM1: Nucleophosmin
PD-L1: Programmed cell death 1 ligand 1
TNBC: Triple-negative breast cancer
NAT10: N-acetyltransferase 10
IFN-γ: Interferon gamma
CTLA-4: Cytotoxic T-lymphocyte associated protein 4
TIL: Tumor-infiltrating lymphocytes
IHC: Immunohistochemical staining
Co-IP: Coimmunoprecipitation
CDK1: Cyclin-dependent kinase 1
CDC25C: Cell division cycle 25C
ac4C: N4-acetylcytidine
SIRT1: Sirtuin 1
CCDC84: Coiled-coil domain containing 84
MORC2: MORC family CW-type zinc finger 2
COL5A1: Collagen type V alpha 1 chain
KIF23: Kinesin family member 23
PDX: Patient-derived tumor xenograft
HNSCC: Head and neck squamous cell carcinoma
