# Supplementary material for: Targeting the NAT10/NPM1 axis abrogates PD-L1 expression and improves the response to immune checkpoint blockade therapy
Source: Mol Med. 2024 Jan 20;30:13. doi: 10.1186/s10020-024-00780-4 (PMC10799409; doi:10.1186/s10020-024-00780-4)
Supplement: Supplementary file 1 — Additional file 1: Figure S1. A The correlation between NPM1 and PD-L1 (CD274) expression levels in colon cancer and skin cutaneous melanoma in the TIMER 2.0 database. B NAT10 was identified by mass spectrometry. C NPM1 and NAT10 loci were evaluated by IF staining in HCT116 cells. D MDA-MB-231 and HCT116 cells were treated with 25 ng/ml IFN-γ for 24 h, and PD-L1 expression was subsequently evaluated. E PD-L1 expression was measured by western blot (left) and qPCR (right) after NAT10 was knocked down by siRNA in A375 cell. Data are presented as the mean ± s.d. of three independent experiments. ****P < 0.0001. Figure S2. A The acetylation sites of NPM1 were detected by mass spectrometry. B PD-L1 expression was measured by western blot (right) and qPCR (left) after the indicated plasmids were transfected into MDA-MB-231 cell. C Gating strategies used for flow cytometric analyses in mouse tumor tissues. D The tumor volume of every mouse in each group (n = 8) was recorded twice a week. Data are presented as the mean ± s.d. of three independent experiments. *P < 0.05, **P < 0.01; ns, not significantly different. Figure S3. A Representative images of IHC staining of NAT10 (left) and PD-L1 (right) in 40 TNBC patient tissues. B Correlation analysis between NAT10 expression and PD-L1 expression was performed in 85 colon cancer patients using two-tailed Pearson’s chi-square test. (C) PDL1 expression was measured by western blot after HCT116 cells were treated with CPTH2 for 48 h. Table S1. Multivariate analysis for OS in 85 colon cancer patients. Table S2. Correlation analysis of NAT10 expression and clinical features in 85 colon cancer patients. [file 10020_2024_780_MOESM1_ESM.pdf]

Figure S1

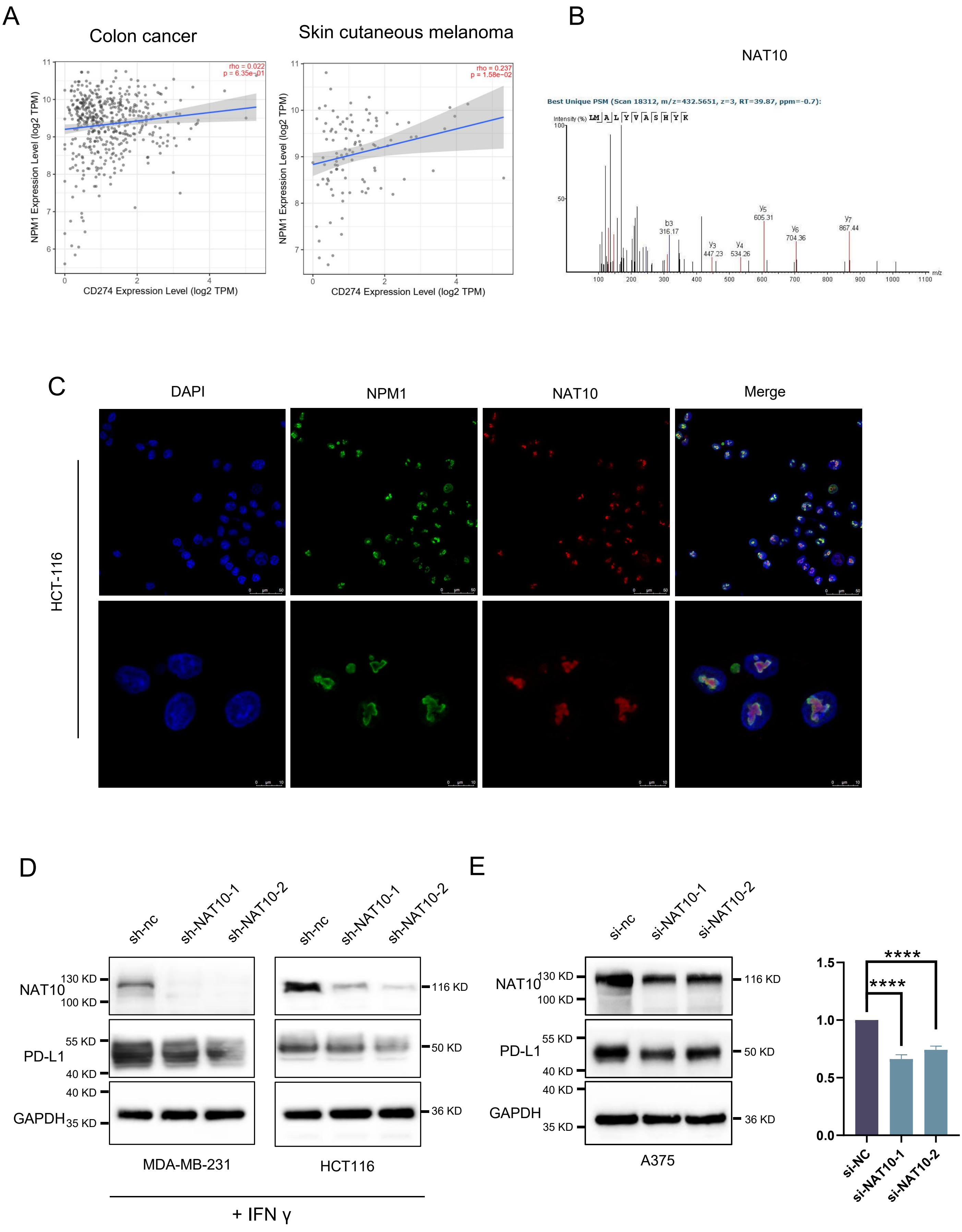
A
Colon cancer
Skin cutaneous melanoma
rho = 0.022
p = 6.35e-01
rho = 0.237
p = 1.58e-02
NPM1 Expression Level (log2 TPM)
CD274 Expression Level (log2 TPM)
B
NAT10
Best Unique PSM (Scan 18312, m/z=432.5651, z=3, RT=39.87, ppm=-0.7):
Intensity (%)
LMALYVASHYK
b3 316.17
y3 447.23
y4 534.26
y5 605.31
y6 704.36
y7 867.44
m/z
C
DAPI
NPM1
NAT10
Merge
HCT-116
D
sh-nc
sh-NAT10-1
sh-NAT10-2
NAT10
PD-L1
GAPDH
130 KD
100 KD
55 KD
40 KD
35 KD
116 KD
50 KD
36 KD
MDA-MB-231
HCT116
+ IFN γ
E
si-nc
si-NAT10-1
si-NAT10-2
A375
****
si-NC

Figure S2

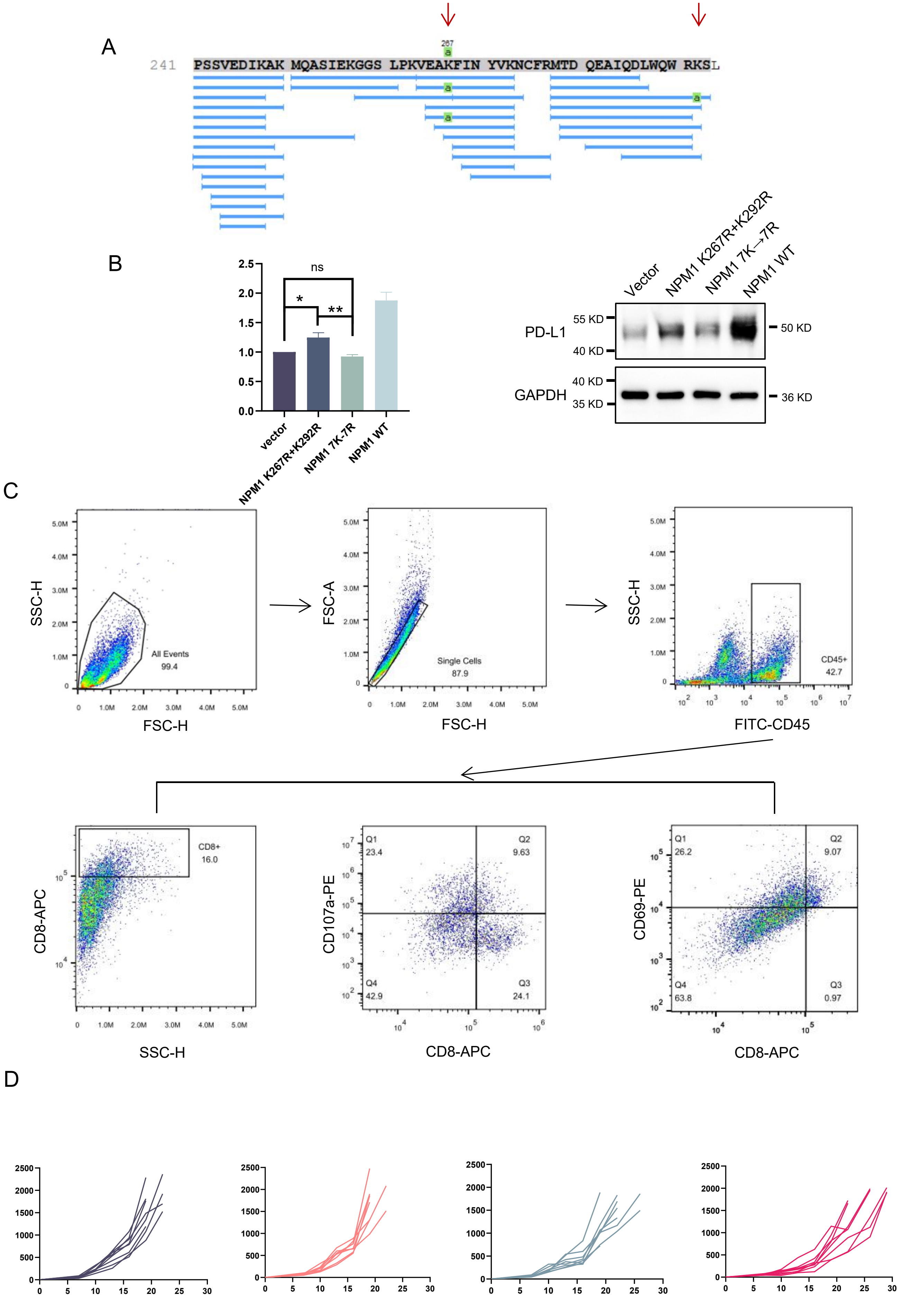

A
267
241 PSSVEDIKAK MQASIEKGGS LPKVEAKFIN YVKNCFRMTD QEAIQDLWQW RKSL
B
ns
*
**
2.5
2.0
1.5
1.0
0.5
0.0
vector
NPM1 K267R+K292R
NPM1 7K-7R
NPM1 WT
Vector
NPM1 K267R+K292R
NPM1 7K→7R
NPM1 WT
PD-L1
55 KD
40 KD
50 KD
GAPDH
40 KD
35 KD
36 KD
C
SSC-H
FSC-H
All Events
99.4
FSC-A
Single Cells
87.9
FITC-CD45
CD45+
42.7
CD8-APC
SSC-H
CD8+
16.0
CD107a-PE
Q1
23.4
Q2
9.63
Q4
42.9
Q3
24.1
CD69-PE
Q1
26.2
Q2
9.07
Q4
63.8
Q3
0.97
D
2500
2000
1500
1000
500
0
0
5
10
15
20
25
30

Figure S3

A

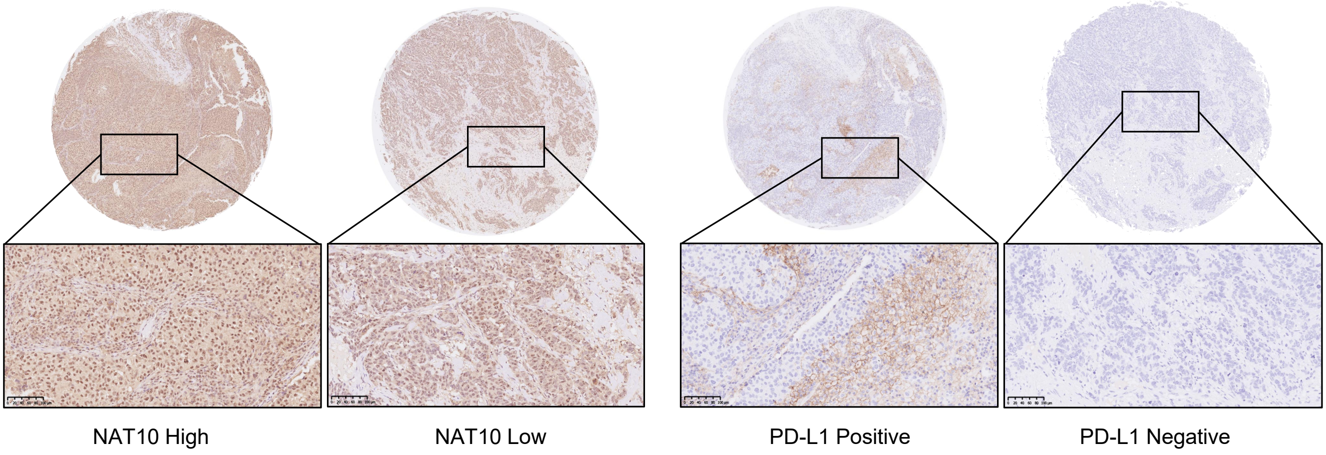

B

	NAT10 Expression		
	Low	High	Total
**PD-L1 Expression**			
**Negative**	18	51	69
**Positive**	2	14	16
**Total**	20	65	85

P=0.338 R=0.125

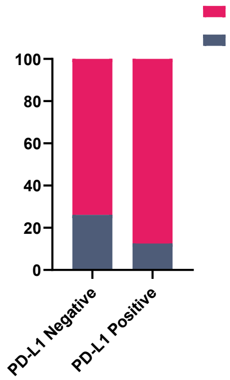

C

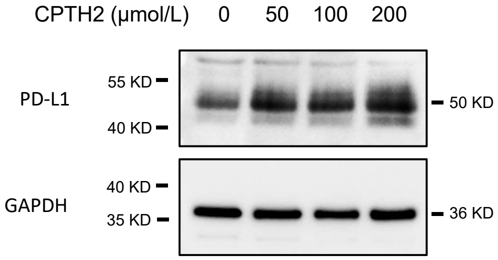

Figure S1: (A) The correlation between NPM1 and PD-L1 (CD274) expression levels in colon cancer and skin cutaneous melanoma in the TIMER 2.0 database. **(B)** NAT10 was identified by mass spectrometry. **(C)** NPM1 and NAT10 loci were evaluated by IF staining in HCT116 cells. **(D)** MDA-MB-231 and HCT116 cells were treated with 25 ng/ml IFN-γ for 24 hours, and PD-L1 expression was subsequently evaluated. **(E)** PD-L1 expression was measured by western blot (left) and qPCR (right) after NAT10 was knocked down by siRNA in A375 cell. Data are presented as the mean ± s.d. of three independent experiments. ****$P < 0.0001$.

Figure S2: (A) The acetylation sites of NPM1 were detected by mass spectrometry. **(B)** PD-L1 expression was measured by western blot (right) and qPCR (left) after the indicated plasmids were transfected into MDA-MB-231 cell. **(C)** Gating strategies used for flow cytometric analyses in mouse tumor tissues. **(D)** The tumor volume of every mouse in each group (n=8) was recorded twice a week. Data are presented as the mean ± s.d. of three independent experiments. *$P < 0.05$, **$P < 0.01$; ns, not significantly different.

Figure S3: (A) Representative images of IHC staining of NAT10 (left) and PD-L1 (right) in 40 TNBC patient tissues. **(B)** Correlation analysis between NAT10 expression and PD-L1 expression was performed in 85 colon cancer patients using two-tailed Pearson's chi-square test. **(C)** PD-L1 expression was measured by western blot after HCT116 cells were treated with CPTH2 for 48 hours.

table S1: Multivariate analysis for OS in 85 colon cancer patients. .

Variables	Multivariate			
		95% CI		
	**HR**	**Lower**	**Upper**	**P-value**
**Age (continue)**	0.999	0.964	1.035	0.949
**Primary tumor location (left vs. right)**	2.507	0.879	7.153	0.086
**Clinical stage (I vs. II vs. III vs. IV)**	2.493	1.146	5.423	**0.021**
**Sex (female vs. male)**	0.718	0.244	2.133	0.547
**Tumor thrombus (pos vs. neg)**	4.751	1.866	12.097	**0.001**
**Nerve invasion (pos vs. neg)**	1.098	0.349	3.454	0.872
**BRAF mutation (pos vs. neg)**	1.682	0.131	21.588	0.690
**RAS mutation (pos vs. neg)**	1.043	0.365	2.984	0.937
**NAT10 status (high vs. low)**	2.737	0.431	17.389	0.286

Data was analyzed by Likelihood Ratio (LR) text.

table S2: Correlation analysis of NAT10 expression and clinical features in 85 colon cancer patients.

Variable	NAT10 expression （n(%)）		P-value
	Low	High	
**Age (continue)**	62 (43~81)	57.5(29~86)	
**Primary tumor location**			
**left**	10（25.0）	30（75.0）	1.000
**right**	9（23.7）	29（76.3）	
**Clinical stage**			
**I**	5（55.6）	4（44.4）	**0.036**
**II-III**	15（20.8）	57（79.2）	
**IV**	0（0.00）	4（100）	
**Sex**			
**female**	6（13.6）	38（86.4）	**0.040**
**male**	14（34.1）	27（65.9）	
**Tumor thrombus**			
**positive**	6(21.4)	22(78.6)	1.000
**negative**	14(24.6)	43(75.4)	
**Nerve invasion**			
**positive**	2(12.5)	14(87.5)	0.338
**negative**	18(26.1)	51(73.9)	
**BRAF mutation**			
**positive**	1（50）	1（50）	0.358
**negative**	14（18.9）	60（81.1）	
**RAS mutation**			
**positive**	8（22.9）	27（77.1）	0.825
**negative**	10（20.8）	38（79.2）	

Data was analyzed by two-side Pearson Chi-square test.
